# Relationship between resilience at work, work engagement and job satisfaction among engineers: a cross-sectional study

**DOI:** 10.1186/s12889-024-18507-9

**Published:** 2024-04-18

**Authors:** Bassma Abdelhadi Ibrahim, Sarah Mohamed Hussein

**Affiliations:** Department of Public Health, Community Medicine, Environmental Medicine, and Occupational Medicine, Faculty of Medicine, Suez Canal University, Ismailia, Egypt

**Keywords:** Engineers, Resilience, Work engagement, Vigor, Absorption, Dedication, Job satisfaction

## Abstract

**Background:**

Workplace challenges can negatively affect employees and the organization. Resilience improves work-related outcomes like engagement, satisfaction, and performance. Gaps exist in studying resilience at work, particularly in relation to engagement and satisfaction. Therefore, this study aims to investigate relationship between Resilience at Work, Work Engagement and Job Satisfaction among engineers in an Egyptian Oil and Gas Company.

**Methods:**

It was a cross-sectional study. The target population was the engineers who are working in Egyptian Oil and Gas Company. The study was performed on 100 engineers. Participants were enrolled by simple random sampling technique via an online questionnaire. The study was conducted from May 2023 to the end of September 2023. The data were collected in the duration of June to August 2023. Data was obtained through a structured and personally accomplished questionnaire, which was disseminated electronically via email. The questionnaire comprises of personal information, work experience, a Resilience at Work scale consisting of 20 items, the Utrecht Work Engagement Scale with nine items to evaluate work engagement, and the 20-item Short-Form Minnesota Satisfaction Questionnaire was utilized to determine employee satisfaction. The bivariate analysis employed independent samples t-test and Mann-Whitney U test. The associations between scores were measured by Spearman rho correlation. Simple linear and multiple linear regressions were used to predict work engagement and job satisfaction.

**Results:**

A statistically strong positive correlation was observed among all the aspects of work engagement, including vigor, absorption, and dedication. This study demonstrated a significant correlation between resilience and work engagement (*r* = 0.356, *p* < 0.05). There was a strong correlation between resilience and job satisfaction (*r* = 0.608, *p* < 0.05). A significant moderate correlation was determined between job satisfaction and work engagement (*r* = 0.396, *p* < 0.05). Both gender with a female coefficient of -15.517, and resilience with a coefficient of 0.235 significantly predicted work engagement. Whereas, the significant predictors of job satisfaction were resilience (β = 0.294), and work engagement (β = 0.283).

**Conclusions:**

Resilience greatly affects work engagement and job satisfaction. Thus, organizations need to promote resilience in employees to create a positive work environment and increase productivity.

## Background

Resilience has become an essential element in the success and well-being of employees in today’s fast-paced and demanding work environment. This is especially evident in high-pressure industries like oil and gas sector. Most oil and gas industry workers experience various stressful conditions and encounter numerous challenges and pressures in their daily work, impacting their health [1–3].

The Oil and Gas industry is widely recognized for its challenging and hazardous work environment in terms of safety and occupational risks. Consequently, employees in this field especially engineers frequently encounter intricate obstacles such as working under immense pressure, complying with strict safety protocols, meeting tight project timelines, and keeping up with evolving technologies and market dynamics. An Egyptian survey conducted on a group of 409 workers in the oil and gas industry showed that the work environment had a high level of psychosocial hazards, as well as mild levels of anxiety and moderate levels of depression and stress [4]. Similar studies in Nigeria and Iran also found high levels of occupational stress among employees in the Oil and Gas industry [5, 6]. One important factor that has been found to be crucial to deal with these challenges and stress is the development of resilience and positive psychological well-being among employees. This is necessary to ensure operational efficiency, safety, and overall wellness for professionals in the industry.

Resilience is commonly referred to as the ability to recover from adversity, conflict, or failure. It can also apply to positive events, progress, and increased responsibilities. So, resilient employees have better awareness and ability to be more flexible, improvise, and adjust quickly to change [2]. Resilience has a positive impact on work outcomes like engagement, satisfaction, and performance [7–9]. Resilient personnel could create a problem-solving pattern that allows them to contribute best to their workplace. Also, resilient individuals are successful in dealing with workplace adversity, producing persistent and favorable work attitudes leading to engagement [10].

Work engagement is a state of mind that involves concentration, energy, and enthusiasm in one’s work. It is described as being vigorous, dedicated, and absorbed. It is beneficial for both individuals and organizations as it promotes motivation and commitment [10, 11]. In Indonesia, a study conducted among 205 respondents working as merchandisers in Fast Moving Consumer Goods field under outsourcing companies demonstrated a positive association between employee resilience and work engagement (*r* = 0.346, *p* < 0.01). The findings showed that employees who possess high resilience levels tend to exhibit greater work engagement [12]. Another study by Aggarwal (2022) unveiled a significant correlation between resilience and work engagement among employees (*r* = 0.024, *p* < 0.05) suggesting that resilience and work engagement are interrelated and have a mutual impact on each other [13]..

Furthermore, resilience not only serves as a protective factor, but it can also influence employee job satisfaction. Job satisfaction can be defined as a positive feeling about one’s job as an outcome of an individual’s perception and evaluation of his work. Its level is closely associated with employee motivation and productivity [14]. An Iranian study conducted among employees of an Iranian petrochemical company revealed that the level of employees’ job satisfaction was moderate [6]. Also, a research conducted by Bernard (2021) aimed to investigate the connections between resilience, job satisfaction, and anticipated turnover among chief nursing officers throughout the United States and found a significant link between resilience and job satisfaction, with a positive correlation coefficient of 0.28 [15].

While there is existing literature on resilience, work engagement, and job satisfaction in various industries, there is a significant research gap in specifically addressing these constructs within the unique context of the oil and gas industry. Furthermore, in this context understanding engineers’ resilience, work engagement, and job satisfaction as well the factors that contribute to them is essential for maintaining a highly skilled and motivated workforce [2]. So, this study aims to investigate the complex relation between resilience, work engagement, and job satisfaction among engineers working in the oil and gas company with core concerns revolving around understanding how resilience affects work engagement and job satisfaction, and how these relationships manifest within this organizational setting. This study holds significant implications for both organizational leaders and employees. By gaining insights into the interplay of these constructs, organizations can develop targeted interventions and strategies to enhance employee well-being and performance, leading to a more resilient and satisfied workforce. The subsequent sections of this research will begin by the methodological approach employed in this study. Following this, the findings and their implications will be discussed, concluding with recommendations for future research and practical applications.

## Methods

### Study design

It was a cross-sectional study to examine the relation between workplace resilience, work engagement and job satisfaction. The study was conducted from May 2023 to the end of September 2023. The data were collected in the duration of June to August 2023.

### Population

The study was carried out on engineers working in an Egyptian Oil and Gas Company. Among the 3,000 employees working in the Egyptian Oil and Gas Company, 500 were engineers.

#### Inclusion criteria

Both males and females with the job title “engineer” and who graduated from the faculties of Engineering, Science, and Computers and Information were eligible to participate in the study.

#### Exclusion criteria

New engineers hired for less than a year, part-time engineers were excluded from the population.

### Sample size

G*Power 3.1.9.7 software calculated sample size using exact test family, two tails, and the α error was determined at 0.05 and power = 0.80, r = correlation ρ H1 was determined twice based on correlation between resilience at work and job satisfaction (*r* = 0.28) [15],, and correlation between resilience and work engagement (*r* = 0.346) [12, 16].

After estimation of the sample size for each outcome, the largest sample size = 97 participants. We added 10% of the sample size to adjust for non-response, so the sample size was raised into108 participants. The questionnaire was sent to those engineers, only 100 engineers responded and agreed to participate in the study which covers the required sample. So, the final recruited number of participants were 100 engineers, which represents 92.5% response rate.

### Sampling technique

Engineers were recruited into the study by simple random sampling. A sampling frame of all eligible engineers was formulated by contacting the human resources department. By random generator of SPSS software program version 22, the authors selected the chosen engineers. Through the technology information department, the authors received the email addresses of the engineers. The authors sent invitations to the chosen engineers including the titles of the study, its purpose researchers’ contact information, and informed consent. By accepting the informed consent, the respondents took part in the research.

### Tool of data collection

We gathered data by using a well-structured and self-administered questionnaire. The structure of the study consisted of four distinct sections. Three tools used to assess resilience, work engagement and job satisfaction are valid and reliable tools [17–19]. The initial section encompassed personal data, while the second section evaluated resilience by using the Resilience at Work (RAW) scale created by Winwood et al. (2013) [17]. This scale consisted of 20 items and employed a seven-point Likert scale for rating. The scores on the scale ranged from 1, indicating strong disagreement, to 7, indicating strong agreement. It is reliable instrument as the calculated Cronbach’s alpha = 0.94.

The third section explored work engagement, utilizing the nine-item Utrecht Work Engagement Scale (UWES) established by Schaufeli et al. (2006) [18]. This scale encompassed the three aspects of work engagement: vigor, absorption, and dedication. The scoring of responses is done on a 6-point Likert scale that ranges from ‘0’ (never) to ‘6’ (always), with a reliability coefficient of α = 0.96. Lastly, the assessment of job satisfaction involved the utilization of the Minnesota Satisfaction Questionnaire (twenty-item Short-Form) (MSQ). The items on this scale were rated on a five-point Likert scale, with 1 indicating very dissatisfied and 5 indicating very satisfied. Item responses were aggregated to create a total score, where lower scores indicated lower levels of job satisfaction [19]. The MSQ is a reliable questionnaire whereas the calculated Cronbach’s alpha for MSQ was 0.91.

The original questionnaire in English was bidirectionally “back–back” translated into Arabic. The English-to-Arabic translation was first done by a bilingual translator. Face validity of the Arabic translated version was tested whereas it was reviewed by another bilingual translator for accuracy. Discrepancies resolved through discussion. The questionnaire was back-translated from Arabic to English by a third translator. Adjustments are made to ensure meaning is preserved. A pilot study was carried out on 10 engineers to test the questionnaire to ensure language clarity and feasibility. Data from the pilot study was excluded from the final analysis. After performing any modification in the question’s language according to the pilot participants’ response. The final form of the translated questionnaire was distributed. It was an online Google form survey that was sent to the employees through their emails. The researchers will obtain the participants’ informed consent before starting to fill out the questionnaire.

#### Data management

The SPSS software program version 22 was utilized for data entry and statistical analysis. Qualitative variables were described in frequency and percentage form. While quantitative variables were summarized in the form of mean (standard deviation) or median (interquartile range). The normality of continuous data was tested by Kolmogorov-Smirnov test. The bivariate analysis employed independent samples t-test and Mann-Whitney U test. The associations between resilience, work engagement and job satisfaction scores were measured by Spearman rho correlation. Simple linear and multiple linear regressions were used to predict work engagement and job satisfaction. A significance level of *p*-value less than 0.05 was used to determine statistical significance.

## Results

This work has been carried out on 100 engineers working in Egyptian Oil and Gas Company. Table 1 displays the participants’ characteristics. Most were male (87%) and married (87%), had a university education (88%), and lived in urban areas (88%). The average work experience was 14.34 ± 5.93 years. Table 1 also shows the scores for resilience, work engagement, and job satisfaction: 109.25 ± 18.97, 39.82 ± 12.41, and 79.53 ± 11.67, respectively. The scores for vigor, absorption, and dedication were 12.88 ± 4.14, and 13.40 ± 4.38, 13.54 ± 4.50, respectively.

**Table 1:** Participants characteristics (*n* = 100)

Participants Characteristic	No.	%	
**Gender**			
Female	13	13.0	
Male	87	87.0	
**Age**			
Mean (SD)	39.02 (6.92)		
Range	26–58 years		
Median (IQR)	38 (8.75)		
**Age Group**			
26–38 years	56	56	
> 38–58 years	44	44	
**Marital Status**			
Unmarried	13	13.0	
Married	87	87.0	
**Educational Level**			
University	88	88.0	
Master or doctorate	12	12.0	
**Residence**			
Urban	88	88.0	
Rural	12	12.0	
**Duration of work experience** Mean (SD)	14.34 (5.93)		
Median (IQR)	13.50 [10]		
**Years of experience** (less than 15 years)	54		54
≥ 15 years	46		46
**Resilience score** mean (SD)	109.25 (18.97)		
**Work engagement score** mean (SD)	39.82 (12.41)		
**Work engagement subscales** mean (SD)			
Vigor	12.88 (4.14)		
Dedication	13.54 (4.50)		
Absorption	13.40 (4.38)		
**Job satisfaction score** mean (SD)	79.53 (11.67)		

SD: Standard Deviation, IQR: Interquartile Range

**Fig. 1:**
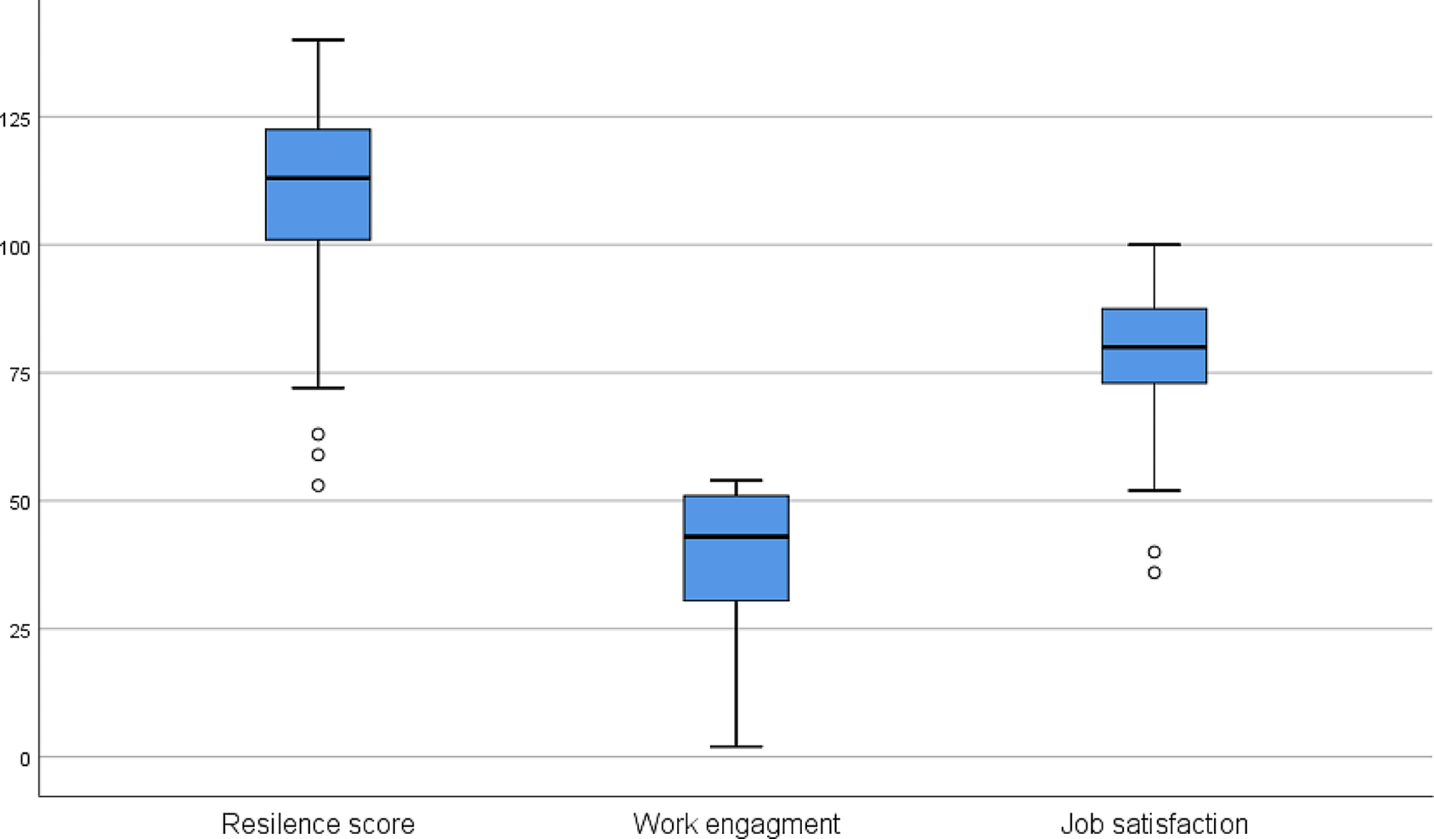
Box-and-whisker plot of resilience, work engagement, and job satisfaction scores (*n* = 100)

As seen in Fig. 1, the median resilience score was 113.00 and the interquartile range (IQR) was 21.75. While the median work engagement score was 43.00 and the IQR was 20.75. However, the job satisfaction median and IQR were 80.00 and 14.75; respectively.

Table 2 summarizes the relation between the sociodemographic characteristics and the 3 parameters of the study. By using Mann-Whitney U test, the only significant factor was the gender for work engagement, whereas the work engagement was significantly higher among males 41.95 (11.54) than females 25. 54 (8.00).

**Table 2:** Relation between sociodemographic characteristics and resilience, work engagement and job satisfaction (*n* = 100)

Variables	Resilience Mean (SD)	Work engagement Mean (SD)	Job satisfaction Mean (SD)
**Gender**			
(Male)	109.75 (18.86)	41.95 (11.54) *	79.58 (11.82)
Female	105.92 (20.16)	25. 54 (8.00)	79.23 (11.04)
**Age Group**			
26–38 years	108.73(19.59)^a^	39.54 (11.03)^a^	79.43 (12.12)^a^
> 38–58 years	109.91 (18.36)	40.18 (14.10)	79.66 (11.21)
**Marital Status** (Unmarried)	111.15 (13.86)	39.92 (8.64)	76.77 (6.54)
Married	108.97 (19.67)	39.81 (12.92)	79.95 (12.23)
**Educational Level** (University)	109.72 (18.34)	40.00 (11.96)	79.42 (11.05)
Master or doctorate	105.83 (23.777)	38.50 (15.90)	80.33 (16.11)
**Years of experience** (less than 15 years)	106.94 (19.53) ^a^	38.65 (11.28) ^a^	78.37 (11.84) ^a^
≥ 15 years	111.96 (18.13)	41.20 (13.62)	80.89 (11.45)
**Residence** **(**Urban)	109.05 (18.51)	39.77 (12.37)	79.67 (10.70)
Rural	100.75 (22.96)	40.17 (13.27)	78.50 (17.89)

Mann-Whitney U test was used, ^a^ Independent-Samples t test, SD: Standard Deviation, *: Significant *p*-value is less than 0.05

As shown in Table 3, there were significant correlations observed between resilience, work engagement, job satisfaction, and the subscales of work engagement such as vigor, dedication, and absorption. The correlation between resilience and work engagement was found to be significantly positive with a moderate correlation coefficient of rho = 0.356. Similarly, the correlation between work engagement and job satisfaction was also significant with a correlation coefficient of rho = 0.396. Furthermore, there was a strong correlation observed between resilience and job satisfaction (rho = 0.608). Additionally, all the subscales of work engagement showed a significant positive strong correlation.

**Table 3:** Spearman rho correlation matrix of resilience, work engagement, and job satisfaction scores (*n* = 100)

Scores	Resilience	Vigor	Dedication	Absorption	Work engagement
Resilience					
**Vigor**	0.393*				
**Dedication**	0.296*	0.865*			
**Absorption**	0.286*	0.835*	0.885*		
**Work engagement**	0.356*	0.949*	0.958*	0.941*	
**Job satisfaction**	0.608*	0.416*	0.341*	0.322*	0.396*

*: Significant *p*-value is less than 0.05

Table 4 demonstrates the significant predictors of work engagement by simple linear and multiple linear regressions as follows: gender, (female coefficient = -16.416, 95% CI= -23.00 - -9.83, -15.517, 95% CI=-21.597 - -9.436; respectively), resilience (coefficient = 0.254, 95% CI = 0.133–0.375, 0.235, 95% CI= -21.597- -9.436; respectively).

**Table 4:** Linear regression of work engagement predictors (*n* = 100)

Variables	Work engagement					
	Univariate regression			Multivariate regression		
	Coefficient	95% CI of Coefficient	*P* value	Coefficient	95% CI of Coefficient	*P* value
Gender (females)	-16.416	-23.00, -9.83	0.000*	-15.517	-21.597, -9.436	0.000*
Resilience	0.254	0.133, 0.375	0.000*	0.235	0.127, 0.343	0.000*

R^2^ of multivariate model = 0.573, *: Significant *p*-value is less than 0.05

With regards to job satisfaction predictors, as seen in Table 5, resilience and work engagement were significant predictors, resilience coefficient by univariate analysis was 0.366, 95% CI = 0.266–0.465, and by multivariate regression was 0.294, 95% CI = 0.192–0.395. Moreover, the coefficients of work engagement were 0.457, 95% CI = 0.293–0.622 and 0.283, 95% CI = 0.128–0.438 by univariate and by multivariate analyses respectively.

**Table 5:** Linear regression of job satisfaction predictors (*n* = 100)

Variables	Job satisfaction					
	Univariate regression			Multivariate regression		
	Coefficient	95% CI of Coefficient	*P* value	Coefficient	95% CI of Coefficient	*P* value
Resilience	0.366	0.266, 0.465	0.000*	0.294	0.192, 0.395	0.000*
Work engagement	0.457	0.293, 0.622	0.000*	0.283	0.128, 0.438	0.000*

R^2^ of multivariate model = 0.418, *: Significant *p*-value is less than 0.05

## Discussion

Engineers in oil and gas industry may face high pressures at work due to ongoing global change, economic recession, and work intensification.These pressures can negatively impact their psychological and physical health, as well as their engagement at work. In this study, we aimed to examine the relation between resilience, work engagement, and job satisfaction among engineers working in an Egyptian gas and oil company.

This study therefore set out in a sample of 100 engineers. The mean age was 39.02 ± 6.92 years. The male gender constituted the majority (87%) of the sample population, while an equal proportion of the sample (87%) were reported to be married, and the sample’s educational attainment was a university education (88%). The mean duration of employment was 14.34 ± 5.93.

Resilience refers to individuals’ ability to effectively handle significant change, adversity, or risk by raising the threshold at which stress arousal occurs [20, 21]. Based on the present study, the mean resilience score among the surveyed engineers was 109.25 ± 18.97. This indicates that, on average, the engineers in our study demonstrated a relatively high level of resilience. In contrast, an Ethiopian study examining burnout and resilience levels among healthcare professionals reported a lower mean resilience score compared to our study’s engineers, with a score of 78.36 ± 17.78 [22]. The difference can be explained by various factors. These factors include differences in the sampled populations, like the specific industry or qualifications of the engineers and the challenges faced by health professionals. Cultural and contextual factors, such as societal norms and work environments, may also have affected resilience levels differently in the two groups. Additionally, Chen et al. (2017) conducted a study on Canadian construction workers and found that higher resilience scores were linked to better stress management abilities at work [23].

Work engagement is made up of three dimensions: vigor, dedication, and absorption. Employees who are engaged demonstrate a high level of energy and mental resilience, and they willingly put in a significant amount of effort into their assigned tasks. Additionally, they express enthusiasm and take pride in their work [24]. In our study, the mean work engagement score among engineers was 39.82 ± 12.41. While, the mean job satisfaction score was 79.53 ± 11.67. Regarding, dimensions of work engagement, the most obvious finding was that vigor was strongly linked to both absorption and dedication (*r* = 0.835, 0.865, *p* < 0.05), respectively. Also, a strong correlation was observed between dedication and absorption (*r* = 0.885, *p* < 0.05). A similar finding has been identified by Abd Elhamed and Hessuin, (2022) who reported a significantly strong positive correlation between all features of work engagement vigor, dedication, and absorption [25].

Another important finding was the presence of a statistically significant and moderate correlation between resilience and work engagement (*r* = 0.356, *p* < 0.05). The multivariate analysis further revealed that resilience significantly predicted work engagement, as indicated by the coefficient value (B = 0.235), indicating that engineers with higher levels of resilience were more likely to experience higher levels of work engagement. It is worth mentioning that vigor, absorption, and dedication exhibited significant correlations with resilience (*r* = 0.393, *r* = 0.286, *r* = 0.296, *p* < 0.05), respectively. This can be clarified by referring to the conservation of resources theory (COR) which focuses on resources and suggests that individuals are motivated to protect and develop their personal resources in order to flourish and deal with stress. Individuals with high levels of personal resources are more likely to show resilience [26]. As a result, resilient individuals are better prepared to handle job demands and setbacks, preserving their resources and maintaining high levels of work engagement. Additionally, resilience can positively affect work engagement as they allow employees to maintain positive attitudes and create conditions that facilitate achieving goals, also enable individuals to appraise themselves and adapt to their environment effectively [10]. This association was confirmed in a study of German healthcare professionals, showing a significant link between resilience and work engagement [27]. Similarly, in a study conducted on 106 South African call center employees, Simons and Buitendach provided evidence of a statistically significant strong correlation between work engagement and resilience (*r* = 0.82, *p* ≤ 0.01). In relation to the subscales of work engagement, They found a statistically significant correlation between vigor and resilience (*r* = 0.48, *p* ≤ 0.01). A statistically significant link was also observed between dedication and resilience (*r* = 0.33, *p* ≤ 0.01), while absorption showed a similar statistically significant correlation with resilience (*r* = 0.34; *p* ≤ 0.01) [28]. In previous studies conducted by Malik and Garg (2018) focusing on Indian employees in the Information technology sector, as well as Abd Elhamed and Hessuin (2022) examining Egyptian nurses, a noteworthy correlation between work engagement and resilience was observed [2, 25].

Contemporary evidence indicates that a significant relationship can be observed between resilience and work happiness, job satisfaction, job performance, and organizational commitment [21, 29, 30]. Interestingly, we also noticed a strong correlation (*r* = 0.608, *p* < 0.05) between resilience and job satisfaction. This implies that as resilience is enhanced, job satisfaction also increases. It is worth noting that also resilience emerged as a significant predictor of job satisfaction (B = 0.294).

One potential reason is that resilience enables the maintenance of effective performance and the ability to deal with challenges. Additionally, it promotes the fulfillment of developmental objectives and is related to mental well-being and overall wellness. Accordingly, highly resilient people have good self-esteem and health and can handle work challenges well, leading to increased efficiency, productivity, and eventually job satisfaction. The relationship could also be clarified using the job demands-resources (JD-R) model. In this model, job characteristics are divided into job demands and resources. Job demands, such as workplace adversity and demands, along with job resources, like resilience, can predict a range of positive and negative job-related outcomes, including burnout and job satisfaction, as well as personal outcomes such as health and well-being [31]. Piotrowski et al. (2022) have employed a similar design to examine resilience, occupational stress, and job satisfaction among nurses and midwives in Poland during the Covid-19 pandemic. According to their findings, there is an average correlation between job satisfaction and resilience (*r* = 0.30, *p* < 0.01). In addition, their study has identified resilience as a statistically significant predictor of job satisfaction (coefficient = 0.17, *p* < 0.001) [14]. Similarly, Srivastava and Madan (2020) have investigated the relationship between resilience and career satisfaction among middle-level managers in private banks in India. They have discovered a significant association between resilience and job satisfaction (B = 0.22, *p* < 0.01) [9]. Kim et al. (2011), Rahmawati (2013), Hudgins (2016), and Ghandi et al. (2017) have all found evidence to support the notion that there is a significant correlation between job satisfaction and resilience. These researchers have reported correlation coefficients of 0.380, 0.366, 0.51, and 0.56, respectively, all of which are statistically significant at *p* < 0.05 [32–35]. Comparably, research conducted in Singapore revealed a strong correlation between resilience and the level of job satisfaction experienced by psychiatric nurses (B = 0.109, *p* = 0.003) [36]. The varying degrees of correlations could be partly related to the nature of the job as well as different tools used to assess job satisfaction and resilience.

This study’s findings further indicated a significant moderate association between job satisfaction and work engagement (*r* = 0.396, *p* < 0.05). In this study, vigor, dedication, and absorption had significantly moderate correlation with job satisfaction (*r* = 0.416, *r* = 0.341, *r* = 0.322, *p* < 0.05), respectively. It is worth noting that work engagement was a significant predictor of job satisfaction (B = 0.283). This implies that engineers who were more involved in their work expressed greater levels of satisfaction with their job. Consequently, these findings highlight the significance of work engagement as a crucial factor in determining job satisfaction. A likely explanation for this might be that work engagement contributes to a sense of accomplishment, experience of personal growth and job enrichment as well as perceiving work as rewarding and meaningful, resulting in job satisfaction. Similarly, job satisfaction enhances motivation, commitment, and enthusiasm toward work, creating a positive environment. It leads to increased work engagement and a sense of belonging. Together, job satisfaction and work engagement create a positive cycle. Our findings align with the work done by Jenaro et al. (2011), which concluded that vigor and dedication were significantly linked to job satisfaction [37]. Similarly, a study in Turkey revealed that work engagement of certified public accountants is positively relate with job satisfaction demonstrating that employee’s higher resilience and work engagement were more satisfied with their job [38]. In line with the findings, a study by Ge et al. (2021) on Chinese healthcare workers also discovered that work engagement and job satisfaction are correlated (*r* = 0.525, *p* < 0.01) [39].

Clearly, it is evident that comparable trends have been identified in numerous sectors in numerous research studies which have demonstrated a positive correlation between resilience, work engagement, and job satisfaction across various occupations. This suggests that these factors play a crucial role in overall job satisfaction and well-being. Moreover, despite the diverse demands and stressors faced by different professions, the overarching theme of the significance of resilience and work engagement in improving job satisfaction remains constant.

Regarding the relationship of sociodemographic and work-related characteristics with resilience, work engagement, and job satisfaction, this study showed that gender specifically being male was significantly associated with higher mean work engagement (*p* < 0.05). Additionally, in this study, the multivariate analysis revealed that among demographic and work-related variables only gender was a significant predictor of work engagement (female coefficient =-15.517). Males may seem more engaged at work for various reasons. Stereotypes and biases linking engineering to masculinity may deter women from pursuing careers in this field. Consequently, there may be fewer female engineers and potentially lower engagement levels among those who do enter the field. Furthermore, Societal expectations and traditional gender roles can hinder women from balancing work and family responsibilities, making it harder for them to fully engage in their careers.

However, this study has been unable to demonstrate associations between other sociodemographic, work-related characteristics, resilience, work engagement, and job satisfaction. The reason for this is not clear but it might be explained in this way; sociodemographic data (e.g., age, gender, education) and work duration may shed some light on individuals’ experiences, but they do not fully determine job satisfaction, resilience, or work engagement. Also, multiple factors, including the nature of the work, work-life balance, relationships with colleagues and supervisors, growth opportunities, and personal characteristics (e.g., psychological factors, emotional intelligence), can influence job satisfaction, resilience, as well as work engagement [40].

The objective of our research was to examine the relationship between resilience, work engagement, and job satisfaction among engineers in an Oil and Gas company. By investigating these relationships, we aimed to provide insights into the factors that contribute to engineers’ job satisfaction within this specific industry context. Our findings successfully addressed this objective and shed light on the interplay between resilience, work engagement, and job satisfaction among engineers. Therefore, the results of our study have important implications for occupational psychology. Our research provides evidence of the relationship between resilience, work engagement, and job satisfaction, which improves our understanding of these concepts. The findings also emphasize the significance of resilience and work engagement in boosting job satisfaction for engineers. Accordingly, it is crucial to the Oil and Gas sector which is a high-pressure industry to implement strategies to support and enhance these factors among its employees by offering opportunities for growth, and creating a supportive work environment.

### Study limitations

While this study sheds light on the link between resilience, work engagement, as well as job satisfaction, several limitations should be acknowledged. Firstly, this study utilized a cross-sectional survey design, which makes it challenging to establish causal relationships among the variables. To address this issue, future research should employ longitudinal designs to examine the temporal relationships between these variables. Secondly, the study was performed within a specific oil and gas company, limiting the generalizability of the findings to other organizations within the industry or to engineers in different sectors. Further research should explore these relationships in diverse companies and industries to provide a broader understanding of the associations between resilience, work engagement, and job satisfaction. Thirdly, the data were gathered through self-report measures, which may introduce information bias. Future research could incorporate objective measures or multiple sources of data to enhance the validity of the findings.

## Conclusions

In conclusion, our study highlights the positive associations between resilience, work engagement, and job satisfaction among engineers within an Oil and Gas company with resilience was a significant predictive factor of both work engagement and job satisfaction. Additionally, this study indicates a significant positive correlation between work engagement and job satisfaction, highlighting the crucial role of resilience and work engagement in fostering job satisfaction among employees.

### Recommendations

Overall, this research strengthens the idea that organizations particularly in the Oil and Gas sector should prioritize initiatives and programs that focus on improving the resilience of their staff members. This can involve providing resources for stress management, workshops or training to enhance resilience, and promoting a culture that values emotional well-being and personal growth. Furthermore, it is recommended that companies actively promote work-life balance in order to assist employees in building and sustaining resilience and engagement at work, with a particular emphasis on female workers. In the future. Longitudinal studies could be conducted to examine the causal relationships between resilience, work engagement, and job satisfaction among engineers in the Oil and Gas industry. Moreover, qualitative research could be employed to delve into the specific mechanisms and factors that contribute to resilience and work engagement within this particular industry. Lastly, it would be beneficial to examine the effects of organizational interventions and support systems on resilience, work engagement, and job satisfaction in order to develop evidence-based practices and policies.

## Data Availability

The datasets utilized and/or analyzed in the present study can be accessed by reaching out to the corresponding author through a reasonable inquiry.

## Abbreviations

RAW: Resilience at Work
UWES: Utrecht Work Engagement Scale
MSQ: Minnesota Satisfaction Questionnaire
SD: Standard Deviation
IQR: Interquartile Range
